# Effect of Bilastine on Diabetic Nephropathy in DBA2/J Mice

**DOI:** 10.3390/ijms20102554

**Published:** 2019-05-24

**Authors:** Roberta Verta, Cristina Grange, Maura Gurrieri, Sara Borga, Patrizia Nardini, Monica Argenziano, Corrado Ghè, Roberta Cavalli, Elisa Benetti, Gianluca Miglio, Benedetta Bussolati, Alessandro Pini, Arianna Carolina Rosa

**Affiliations:** 1 Department of Scienza e Tecnologia del Farmaco, University of Turin, Via P. Giuria 9, 10125 Turin, Italy; robertaverta94@gmail.com (R.V.); maura.gurrieri@live.it (M.G.); sara.borga@edu.unito.it (S.B.); monica.argenziano@unito.it (M.A.); corrado.ghe@unito.it (C.G.); roberta.cavalli@unito.it (R.C.); elisa.benetti@unito.it (E.B.); gianluca.miglio@unito.it (G.M.); 2 Department of Scienze Mediche, University of Turin, C.So Dogliotti 14, 10126 Turin, Italy; cristina.grange@unito.it; 3 Department of Clinical and Experimental Medicine, University of Florence, Viale Pieraccini 6, 50139 Florence, Italy; patrizia.nardini@unifi.it; 4 Department of Biotechnology and Health Sciences, Molecular Biotechnology Center University of Turin, Via Nizza 52, 10125 Turin, Italy; benedetta.bussolati@unito.it

**Keywords:** histamine, histamine H_1_ receptor, kidney, diabetes, slit diaphragm

## Abstract

Diabetic nephropathy is an unmet therapeutic need, and the search for new therapeutic strategies is warranted. Previous data point to histamine H_1_ receptor as a possible target for glomerular dysfunction associated with long term hyperglycaemia. Therefore, this study investigated the effects of the H_1_ receptor antagonist bilastine on renal morphology and function in a murine model of streptozotocin-induced diabetes. Diabetes was induced in DBA2/J male mice and, from diabetes onset (glycaemia ≥200 mg/dL), mice received bilastine (1–30 mg/kg/day) by oral gavage for 14 consecutive weeks. At the end of the experimental protocol, diabetic mice showed polyuria (+195.5%), increase in Albumin-to-Creatine Ratio (ACR, +284.7%), and a significant drop in creatinine clearance (*p* < 0.05). Bilastine prevented ACR increase and restored creatinine clearance in a dose-dependent manner, suggesting a positive effect on glomerular filtration. The ultrastructural analysis showed a preserved junctional integrity. Preservation of the basal nephrin, P-cadherin, and synaptopodin expression could explain this effect. In conclusion, the H_1_ receptor could contribute to the glomerular damage occurring in diabetic nephropathy. Bilastine preserved the glomerular junctional integrity, leading to the hypothesis of anti-H_1_ antihistamines as a possible add-on therapy for diabetic nephropathy.

## 1. Introduction

Diabetic nephropathy (DN) is a common and life-threatening microvascular complication of diabetes mellitus. It affects almost 30–45% of the overall diabetic patients [1,2]. DN is one of the major risk factors for end-stage renal disease (ESRD), cardiovascular diseases, and premature death without progression to ESRD [3,4,5]. Current therapies for DN are aimed to slow disease progression, mainly by ameliorating the glycemic control, inhibiting the renin–angiotensin aldosterone system, and changing the lifestyle [1,6]. However, despite the beneficial effects exerted by these approaches, a large proportion of patients still undergo renal replacement therapy [1,7]. Therefore, further efforts should be done in order to effectively counteract DN.

In the last decades, our understanding of DN has been significantly enhanced. In particular, many events and mediators have been elucidated and clearly implicated in the process leading to the impairment of the glomerular filtration barrier [8]. Among them, abnormal histamine-mediated intercellular signaling has been indicated as a potential component of this process [9,10]. In particular, histamine overproduction was observed in diabetic patients [11]. Moreover, mice deficient in the hystidine-decardoxilase (HDC) enzyme (responsible for histamine synthesis) showed a lower tendency to develop diabetes [12]. Finally, the same overproduction was found in several organs of diabetic rats, with the kidney being the second one in order of histamine increase [13]. Histamine renal content in diabetic condition has been correlated with an increase in HDC enzyme expression and activity [14,15]. Therefore, histamine in the kidney can derive from at least three sources: (i) circulating bad, (ii) local production by epithelial cells, or (iii) infiltrating cells (including mast cells, although their level in the kidney is relatively low), with the intra-renal production being the major contributor [9,10]. Moreover, both in vitro and in vivo studies have provided compelling evidence on the expression and function of histamine receptors in the kidney. Functional histamine receptors in the kidney have a differential distribution along the nephron, with glomerulus expressing H_1_ and H_2_ receptors [16,17,18], proximal tubule H_1_ and H_4_ receptors [19], loop of Henlé H_4_ receptor [20], and distal tubules H_1_ and H_2_ receptors [19]—while the H_3_ receptor was found in the collecting ducts [21].

The cellular expression of H_1_ receptor within glomerulus has been extensively evaluated. At this site, it was found on mesangial cells, where it mediates the histamine-promoted cell contraction [17]. This receptor type was also found on podocyte cell membrane, and its activation—analogous to what was observed for cultured retinal microvascular endothelial cells [22]—was related to the histamine-induced junctional-integrity disruption [18]. Collectively, this evidence indicates a role for the histamine-H_1_ receptor axis in the onset of proteinuria and suggests the hypothesis that histamine affects the glomerular pore density, reducing the filtration surface area and leading to the decrease in the ultrafiltration coefficient [23]. This idea is sustained by the demonstration that H_1_ receptor antagonism decrease proteinuria in a model of anti-glomerular basement membrane (GBM)-induced glomerulosclerosis [24]. In addition, consistent effects were described for (R)-cetirizine in a model of streptozotocin (STZ)-induced diabetes in rats [25]. Interestingly, comparable effects were exerted by both (R)-cetirizine and losartan on proteinuria (Urinary Protein Excretion, UPE; decrease) and creatinine clearance (CrCl; increase). However, given the relatively short extension of the follow-up adopted in this study (eight weeks) with regard to the rate of DN progression [25], these findings cannot be considered conclusive. Further data need to be collected to better understand the pathophysiological and pharmacological role of H_1_ receptor in the context of DN. Therefore, this study aimed to assess the effects of bilastine on renal morphology and function in diabetic DBA2/J mice (the inbred strain of mice most susceptible to develop diabetic nephropathy following STZ administration [26]). Bilastine is a second-generation H_1_ receptor antagonist, currently approved in many countries for the treatment of allergic disorders. Compared with (R)-cetirizine, bilastine is endowed with higher potency with no/minimal affinity for other receptor types [8]. Besides, the pharmacokinetic profile—an oral bioavailability of about 60–90%, the low extension of metabolic clearance, and the long duration of the effect (>24 h) [27,28]—make this drug a good candidate to be studied in a model of DN. Results collected in this study support the hypothesis that the H_1_ receptor blockade reduces the glomerular damage preserving the junctional integrity at the Slit Diaphragm (SD).

## 2. Results

**Figure 1:**
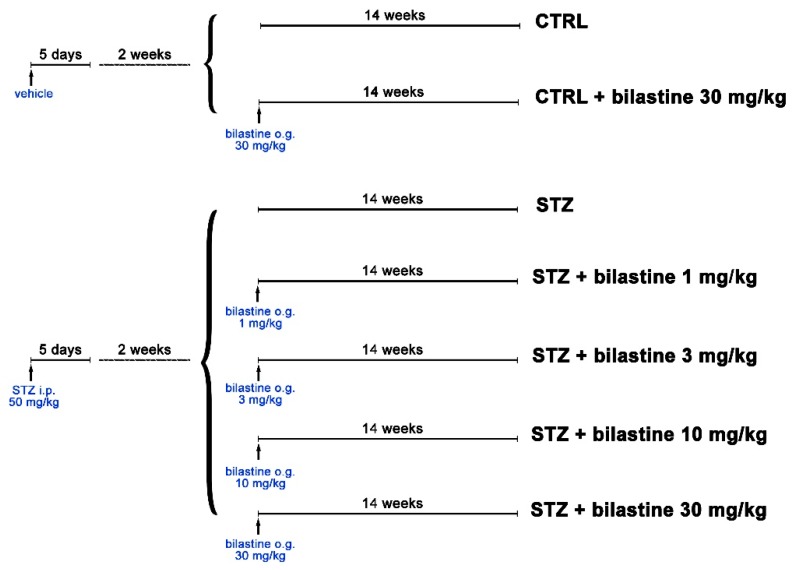
Diagram of the experimental design describing group allocation (*n* = 10 animals/per group). CTRL = control; STZ = streptozotocin; i.p. = intraperitoneal injection; o.g. = oral gavage.

Two weeks after the last STZ injection (Figure 1), 90% of DBA2/J mice developed a diabetic status (≥200 mg/dL) as measured by the 6 h fasting glycaemia. At the end of the study, a severe hyperglycaemia was reached in STZ group (450 ± 56 mg/dL vs. 137 ± 21 mg/dL of the control group). The development of hyperglycaemia was accompanied by glycosuria (Table 1). Bilastine (1, 3, 10, and 30 mg/kg/day by oral gavage from the onset of diabetes; Figure 1) did not significantly affect the hyperglycaemia in non-diabetic animals (147 ± 24 mg/dL) nor in diabetic ones (318 ± 91 mg/dL, 364 ± 74 mg/dL, 293 ± 52 mg/dL, and 319 ± 87 mg/dL, respectively) or glycosuria (Table 1).

**Figure 2:**
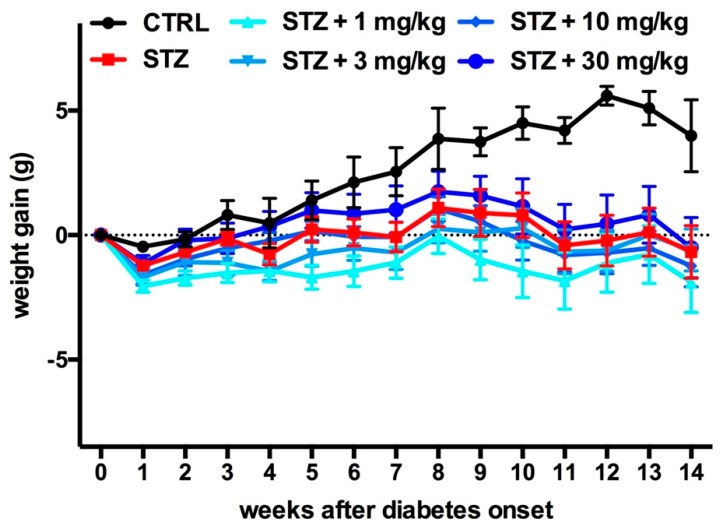
Effect of bilastine on weight gain. Body weight was monitored on a weekly basis, starting from diabetes development (week 0, glycemia ≥200 mg/Dl for 90% of diabetic animals) throughout the experimental period and weight gain was estimated. Data are expressed as mean ± S.E.M. (*n* = 10/group).

Only control animals gained weight, while all diabetic animals displayed significant weight loss over time (Figure 2), even when accounting for differences in food consumption (data not shown). Bilastine did not affect body weight in non-diabetic animals (data not shown) nor in diabetic ones (Figure 2).

### 2.1. Bilastine Effects on Renal Function

For the renal function evaluation, bilastine did not affect any of the measured parameters when administered to non-diabetic animals (Table 1). In comparison with control animals, a significant increase in the 24 h urine volume was measured in STZ-treated animal on week 14 (+196.8%; *p* < 0.05). This change was not prevented by bilastine (Table 1). No sign of infection or obstruction was found, as demonstrated by the negativity for leukocyte presence. In diabetic mice, irrespective of the drug treatment, a trend towards a decrease in the urine pH compared to the control was observed (Table 1). UPE and Albumin-to-Creatinine Ratio (ACR) were significantly increased in diabetic animals compared to the control (*p* < 0.05). The drug was unable to prevent UPE but prevented the development of ACR with a significant effect at the highest dose tested (30 mg/kg). Moreover, a significant drop in CrCl of diabetic mice was measured (Table 1). Bilastine treatment prevented the CrCl reduction in a dose-dependent manner, with the 10 and 30 mg/kg doses restoring CrCl levels to the control (Table 1). Collectively, these data suggest that bilastine could exert a protective effect on renal function. 

### 2.2. Bilastine Effect on Glomerular Structure Alterations

**Figure 3:**
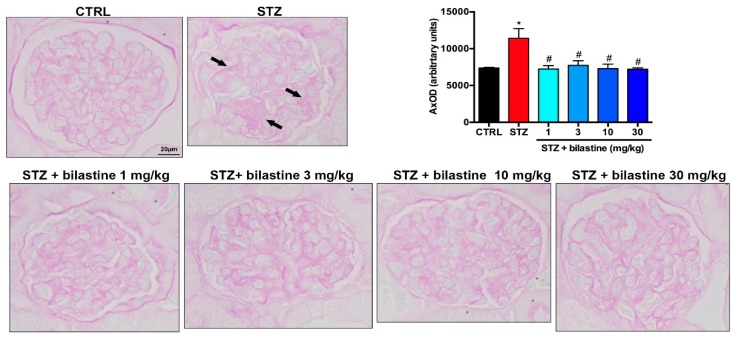
Effect of bilastine on glomerular morphology. Representative micrographs at 100× magnification of PAS stained renal sections. Arrows highlight mesangial matrix expansion. The image is representative of 20 microscopic fields/specimen and 10 animals/group. The densitometric analysis is expressed as the mean ± S.E.M. (*n* = 10); * *p* < 0.05 vs. CTRL; ^#^ *p* < 0.05 vs. STZ.

To assess the effect of bilastine on glomerular structure integrity, the morphological examination of Periodic Acid–Schiff (PAS) staining was performed. The light microscope analysis revealed lobulated glomeruli with moderate mesangial matrix expansion (Figure 3) in the STZ group, thus indicating moderate diabetes-induced damage. No lesions consisting of Kimmelstiel–Wilson nodules were present in the kidneys of diabetic mice. Bilastine administration significantly reduced the mesangial matrix expansion, irrespectively to the dose (Figure 3), thus suggesting that bilastine could prevent glomerular damage.

**Figure 4:**
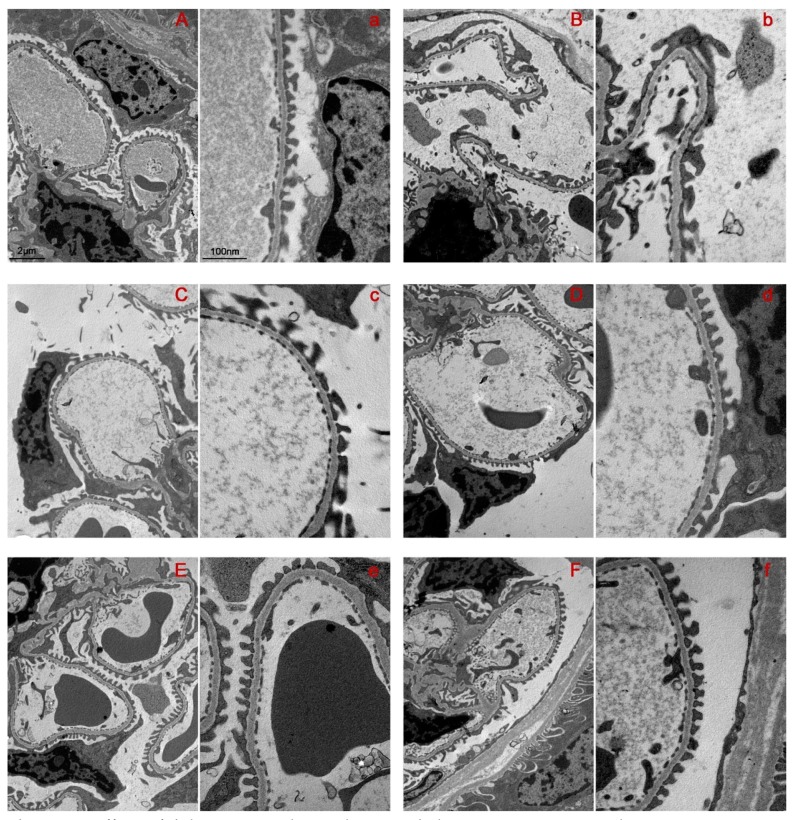
Effect of bilastine on glomerular morphology. Representative electron microscope micrographs showing ultrathin podocyte sections. Micrographs at 10 K (Capital letters) and 25 K (Lower case letter) magnification are representative of 5 animals/group. **A** and **a** = CTRL; **B** and **b** = STZ; **C** and **c** = STZ + bilastine 1 mg/kg; **D** and **d** = STZ + bilastine 3 mg/kg; **E** and **e** = STZ + bilastine 1 mg/kg; **F** and **f** = STZ + bilastine 1 mg/kg.

The ultrastructural evaluation of renal samples is reported in Figure 4. The control group revealed normal glomerular capillary tuft arrangement (Figure 4, Panel A), intact filtration barrier with regular sized, well-aligned podocyte foot processes (FP), and uniform filtration pores (Figure 4, Panel a). In the STZ-induced diabetic mice, the capillary tufts showed irregular fold (Figure 4, Panel B), with areas of the capillary loop surface covered by damaged podocytes. In particular, the FP were diffusely effaced, with irregular size, shape, and variation in the width of the pores (Figure 4, Panels B and b), indicating podocyte loss. No signs of GBM thickening were appreciated in the kidney of diabetic mice. In bilastine treated mice, the architecture of glomerular capillary tuft was preserved (Figure 4; Panels C, D, E, and F). In particular, the drug at 3, 10, and 30 mg/kg was able to structurally preserve the filtration barrier, preventing the effacement of podocyte FP, which appeared to be regular sized and shaped and not detached from the GBM (Figure 4; Panels c, d, e, and f). Also, the observation of filtration pores along the GBM confirmed the protective effect exerted by bilastine. These data show that H_1_ receptor antagonism could preserve the integrity of the filtration barrier.

### 2.3. Bilastine Effect on Slit Diaphragm and Cytoarchitecture Protein Expression

**Figure 5:**
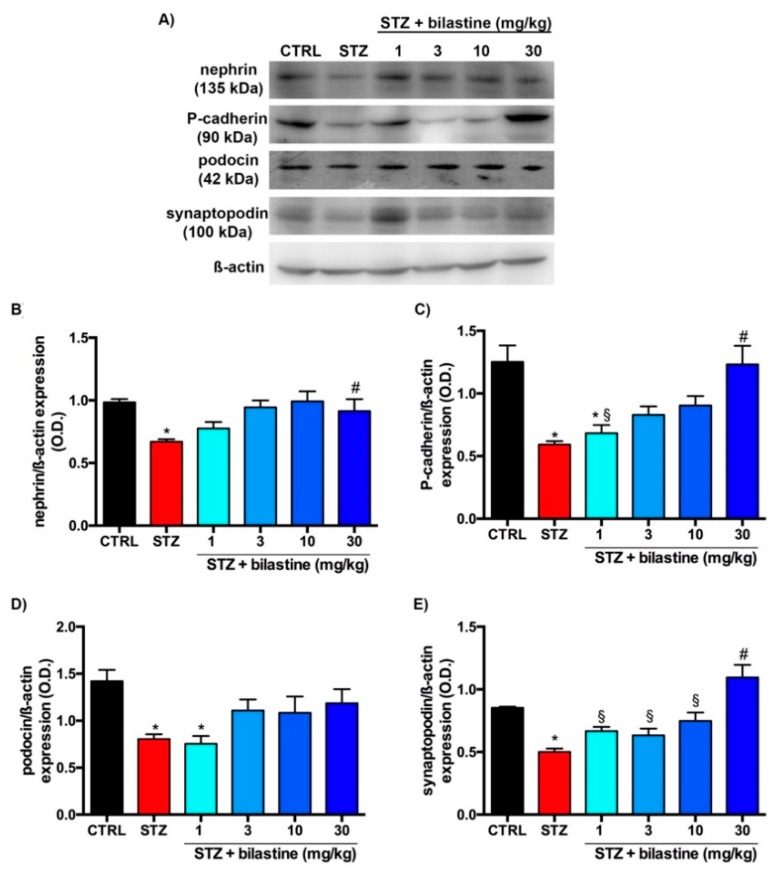
Effect of bilastine on podocyte protein expression. Representative radiograph of nephrin, P-cadherin, podocin, and synaptopodin expression in kidney tissue determined by immunoblotting (**A**). The densitometric analysis of nephrin (**B**), P-cadherin (**C**), podocin (**D**), and synaptopodin (**E**) was performed and expression levels, normalized to β-actin, are expressed as the mean ± S.E.M. of 5 animals/group; * *p* < 0.05 vs. CTRL, ^#^ *p* < 0.05 vs. STZ; ^§^ *p* < 0.05 vs. STZ + bilastine 30 mg/kg.

The effect of bilastine on junctional proteins involved in the maintenance of the SD integrity, nephrin (Figure 5, Panels A and B), P-cadherin (Figure 5, Panels A and C), podocin (Figure 5, Panels A and D) and synaptopodin (Figure 5, Panels A and D), were evaluated by immunoblotting. As expected, diabetic animals showed a down-regulation of all these proteins (Figure 5). Bilastine partially prevented these dysregulations. Indeed, the highest dose was effective in preserving the basal levels of nephrin, P-cadherin and synaptopodin (Figure 5). On the contrary bilastine was not able to prevent podocin loss (Figure 5, Panel D), albeit a trend towards a protective effect was observed. Therefore, the data suggests that bilastine preserves, at least in part, the junctional integrity of the SD.

### 2.4. Bilastine Effect on NHE3 Expression

**Figure 6:**
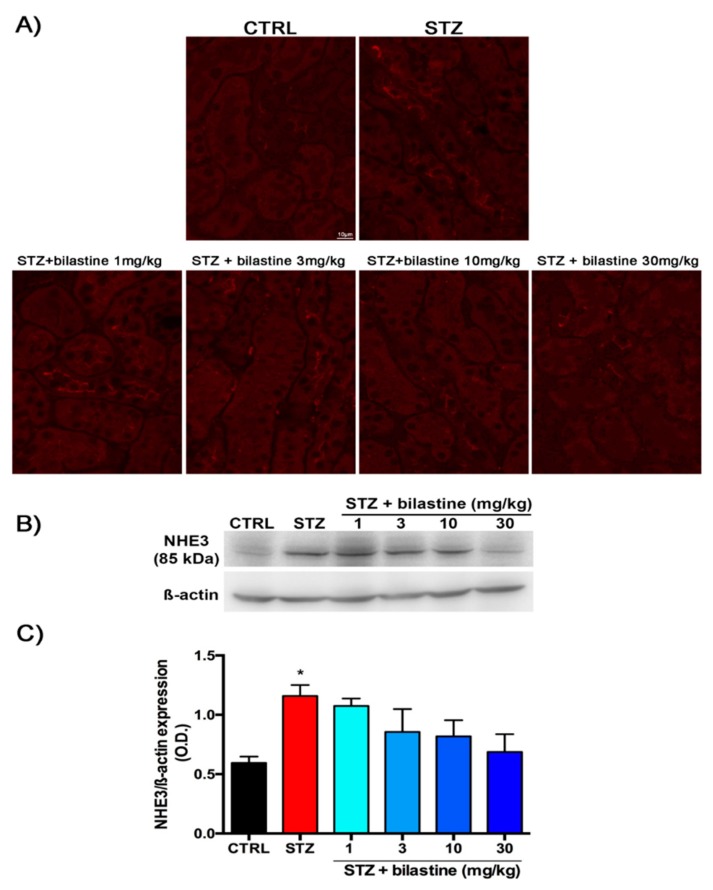
Effect of bilastine on NHE3 tubular expression. Micrographs representative of NHE3 expression on tubular epithelial cells determined by immunofluorescence (63× magnification from 10 animals/group) (**A**). Representative radiograph of NHE3 expression in kidney tissue determined by immunoblotting (**B**). Densitometric analysis of NHE3 expression determined by immunoblotting analysis. Expression levels, normalized to β-actin, are expressed as the mean ± S.E.M. of 5 animals/group; * *p* < 0.05 vs. CTRL, ^#^ *p* < 0.05 vs. STZ (**C**).

In order to better elucidate the effects of bilastine on tubular reabsorption, we investigated the expression of the sodium–hydrogen exchanger (NHE)3 protein expressed on the brush border membrane of renal proximal tubules and responsible for active transcellular reabsorption of NaHCO_3_ and NaCl [29]. The immunofluorescence analysis revealed an increase in the apical expression of NHE3 in diabetic animals compared to the control group. Bilastine-treated mice showed a lower immunopositivity suggestive of a prevention of NHE3 over-expression, but control levels were not restored (Figure 5, Panel A). These data were confirmed by immunoblotting analysis. Diabetic mice displayed a significant up-regulation of NHE3 expression (Figure 6, Panels B and C). Bilastine had no significant effect on NHE3 expression, albeit a trend towards a protective effect was observed.

### 2.5. Bilastine Effect on Tubular Infiltration and Renal Fibrosis

**Figure 7:**
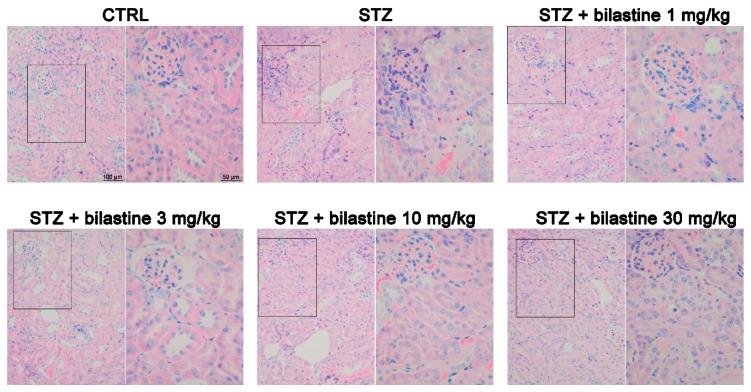
Effect of bilastine on tubular interstitial infiltration. May–Grünwald–Giemsa staining from renal sections. Micrographs at 20× or 40× (insert) magnification are representative of 10 animals/group.

**Figure 8:**
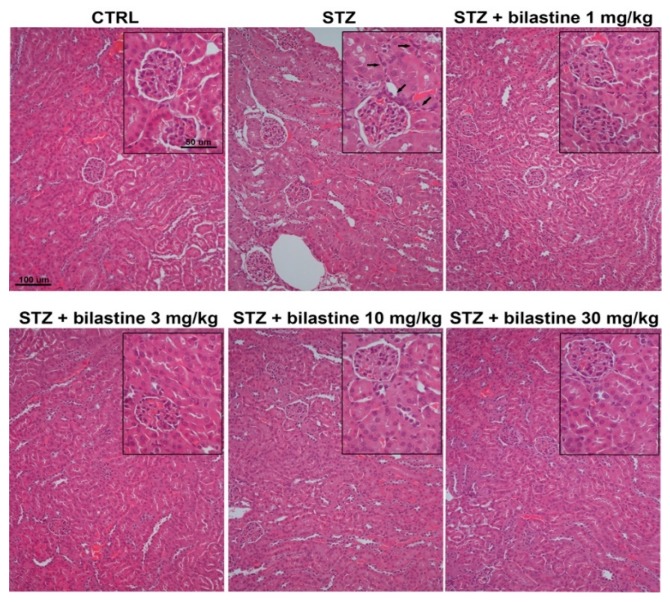
Effect of bilastine on tubular interstitial infiltration. Hematoxylin and eosin staining from renal sections. Arrows highlight infiltrating cells. Micrographs at 20× or 40× (insert) magnification are representative of 10 animals/group.

**Figure 9:**
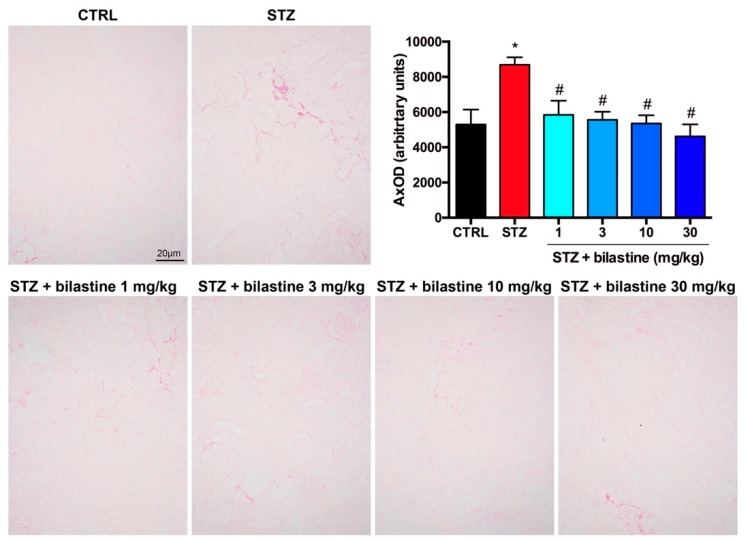
Effect of bilastine on tubular fibrosis. Collagen deposition in the renal interstitium evaluated by picrosirius red stating. Micrographs at 20× magnification are representative of 10 animals/group (20 microscopic fields/specimen). The densitometric analysis is expressed as the mean ± S.E.M. (*n* = 10); * *p* < 0.05 vs. CTRL, ^#^ *p* < 0.05 vs. STZ.

According to the already known pro-inflammatory and profibrotic properties of histamine, and of H_1_ receptor activation, the effect of bilastine on the presence of infiltrating immune cells was evaluated. As shown in Figure 7 and Figure 8, the morphological evaluation of May–Grünwald–Giemsa and hematoxylin and eosin staining showed a moderate iper-cellularity consistent with pro-inflammatory infiltration (leukocyte and neutrophils in particular) in the STZ group. This effect was paralleled by a significant but moderate interstitial fibrosis, as demonstrated by picrosirius red staining (Figure 9). The administration of bilastine reduced the hyper-cellularity (Figure 7) and significantly blunted the collagen deposition induced by STZ-induced hyperglycaemia (Figure 9), irrespective of the dose.

## 3. Discussion

Data reported herein demonstrated that bilastine—preserving the junctional integrity at the glomerular SD—prevents the increase of ACR and the reduction of CrCl in diabetic animals. Interestingly, these data are consistent with previous data by Ichikawa and Brenner (1979) [23] indicating a decrease in the ultrafiltration coefficient following H_1_ receptor activation. In our experimental setting, just a mild renal injury, not recapitulating all the features of DN, was obtained after 14 weeks from the onset of diabetes (≥200 mg/dL in 90% of STZ-treated animals), and a preventive therapeutic approach was used (bilastine was administered as soon as the onset of diabetes). In this model, we obtained a modest matrix mesangial expansion and no thickening of the GBM, however, a consistent podocyte FP effacement and podocyte loss was revealed. The drug prevented these detrimental events.

Interestingly, an apparent discrepancy between functional and morphological data appears. Indeed, while only bilastine 30 mg/kg showed a full protection of renal function, matrix mesangial expansion and collagen deposition were significantly affected by bilastine, irrespective of the dose. However, these are both pro-fibrotic events that could be related to the general anti-inflammatory effect of bilastine, also confirmed in this study in terms of reduction of infiltrating cells. On the contrary, the biochemical evaluation (nephrin, synaptopodin, and P-cadherin expression), keeping with the functional analysis, reached statistical significance only by bilastine at 30 mg/kg. Therefore, we could speculate that histamine is a trigger stimulus for the renal inflammatory response induced by hyperglycaemia, but is also a contributor to the podocyte junctional integrity, to which other mediators, such as angiotensin II [29], participate as well. Consistently, no different effects were shown between (R)-cetirizine and losartan on renal protection in diabetic rats [25].

The observed effect of bilastine is possibly due to the haemodynamic regulation induced by blocking H_1_ receptor. Indeed, histamine can affect glomerular haemodynamics [9,10] mostly through H_1_ receptor [30], and podocytes respond to intracapillary pressure with the loss of the interdigitating foot process pattern [31]. In our model, bilastine was demonstrated to prevent the podocyte FP effacement. This event was sustained by a conserved expression of different proteins involved in the SD maintenance—in particular, the two junctional proteins nephrin and P-cadherin and the actin binding protein synaptopodin. However, all these changes can also be the consequence of a direct effects of bilastine on podocyte, a cell type expressing H_1_ receptor [18]. Indeed, in a previous in vitro study, H_1_ receptor was already demonstrated to modulate P-cadherin expression in human podocytes [18]. In this study, H_1_ receptor activation was demonstrated to generally affect the intra-podocyte junctional machinery and also to down-regulate Zonula Occludens (ZO)-1 expression [18]. Therefore, direct effect(s) on SD protein expression could also occur in vivo. Moreover, a contribution of the H_1_ receptor expressed at the mesangial site [17] cannot be ruled out.

Therefore, even considering the previous data from others, we suggest that H_1_ receptor antagonism could exert a renal protective effect by: (i) The maintenance of glomerular intracapillary pressure [30], (ii) the preservation of constitutive levels of the podocyte junctional-related proteins, and (iii) the prevention of mesangial contraction [17]. These effects are far behind any glycemic control as bilastine, different to (R)-cetirizine [25], was shown to not affect the glycemic status. 

The relationship between histamine signaling and glomerular junctional integrity is an intriguing albeit poorly investigated point. At the molecular level, a plausible link could be found in the role played by Protein Kinases C (PKC) in mediating both the histamine cellular effects and the maintenance of the SD integrity. Indeed, as already reported, H_1_ receptor stimulation induces an increase in inositol 1,4,5-trisphosphate (IP3) second messenger in podocytes [18]. IP3 is known to activate PKC, which plays a crucial role in the preservation of the SD integrity [32]. In particular, PKC is involved in the regulation of SD junctional protein expression, such as P-cadherin [33]. Therefore, we can speculate that an abnormal stimulation of the H_1_ receptors expressed by podocytes could alter the PKC activity, which in turn could contribute to the disruption of the SD integrity. By antagonizing the effects exerted by histamine, bilastine could prevents all these events. Interestingly, no functional effects on renal function were observed when bilastine was administered to non-diabetic mice. Therefore, a possible involvement of inverse agonism of bilastine in the therapeutic effects is unlikely.

The involvement of the histamine H_1_ receptor reported here recalls to the possible analogy between metabolic diseases and allergies [34]. Indeed, the association between the prevalence of Type 1 diabetes and allergic diseases or sensitization [35] has been reported. In homology with asthma, the mechanism(s) underling this association can be related not only to Th2 driven, but also to Th2 non-driven events [34,35]. In both cases, the immuno-metabolic dysfunction is the underlying event. Nevertheless, the release of histamine from mast-cells during an allergic response has been associated to the development of metabolic cardiovascular dysfunctions such as atherosclerosis [36]. Therefore, it could be suggested that the increased histamine levels related to diabetic condition trigger an immuno-metabolic dysfunction, which in turn could contribute to microvascular complications, including the DN.

Considering the data previously obtained on the H_4_ receptor blockade in a similar diabetic model [15], the effects observed at the glomerular level after bilastine treatment are open to many interesting interpretations of the role of histamine in renal pathophysiology, allowing researchers to hypothesize different roles of H_1_ and H_4_ receptors. Indeed, the H_4_ receptor blockade by JNJ39758979 was demonstrated to preserve proximal tubular reabsorption, preventing megalin loss and NHE3 increase in the tubules of diabetic mice. Moreover, water volume was significantly and dose-dependently reduced [15]. These data were consistent with the prevalent H_4_ receptor expression on the tubule [20], especially at the proximal tract [19]. Also, H_1_ receptor is expressed in the tubule [19]. However, bilastine did not prevent the decrease in urine pH and the increase in urine volume or in proteinuria, despite the beneficial effects on ACR and CrCl. Therefore, H_1_ receptor activation could not contribute to the detrimental effects on the tubular reabsorptive machinery, and this hypothesis was confirmed by the effect on NHE3 expression. NHE3 is a proximal tubular transporter, which facilitates sodium reabsorption and proton secretion, thus participating in the acid–base balance [29]. It is known to be up-regulated by a hyperglycaemic status, and its expression is inversely correlated to that of the protein responsible for albumin re-uptake, megalin [37]. In our study, diabetic mice showed a significant NHE3 over-expression compared to the control animals, while bilastine was not effective in preventing NHE3 increase, although a slight but not significant beneficial effect was observed for bilastine at 30 mg/kg. This unexpected result could explain the lack of efficacy on protein excretion and urinary pH value. The high protein outflow and the low pH, even in the presence of bilastine, could account for the observed polyuria.

However, the study has some limitations including: (i) The one end-point design, which does not allow us to evaluate at which stage of DN onset bilastine exerts its effects; and (ii) the administration of bilastine at the early onset of diabetes, and not after DN development. This design configures a preventive approach more than a therapeutic one, and no conclusion on the efficacy of bilastine after the onset of the renal complication can be extrapolated. Therefore, for a final conclusion on the possible therapeutic use of bilastine as an active agent against DN, further studies based on a therapeutic approach (bilastine administration after DN development) are needed.

## 4. Materials and Methods

### 4.1. Materials

All chemicals, not otherwise indicated and rabbit polyclonal anti-β-actin antibody (A2066), were from Sigma Aldrich (St. Louis, MO, USA). The Glucocard MX Blood Glucose Meter was from A. Menarini Diagnostic (Florence, Italy). The Albumin enzymatic immunoassay kits ELISA Quantification Set (E90-134) was from Bethyl Laboratories Inc. (Montgomery, TX, USA). The Urine Strips were from GIMA S.p.a. (Gessate, MI, Italy). The goat polyclonal anti-nephrin antibody (N-20; sc-19000), the goat polyclonal anti-synaptopodin (N-14; sc-21536), rabbit polyclonal anti-P-cadherin (H-105; sc-7893), and rabbit polyclonal anti-podocin (H-120; sc-21009) as well as UltraCruz Autoradiography Film were from Santa Cruz Biotechnology (Dallas, TX, USA); the rabbit polyclonal anti-NHE3 (GTX41967, lot number 821700650) was from Gentex (Santa Antonio, TX, USA). The donkey polyclonal anti-rabbit Fluor 594 AffiniPure (711-585-152) was from Jackson ImmunoResearch Laboratories (Baltimore Pike, West Grove, PA, USA). The rabbit peroxidase-labelled secondary antibody was from Cell Signaling Technology Inc. (Danvers, MA, USA). The BCA™ Protein Assay Kit was from Thermofisher Scientific (Waltham, MA, USA). The Immobilon^®^ PVDF transfer membrane was from Merck Millipore (Milan, Italy). The Acrylamide/Bis solution 29:1 and the Albumin bovine modified Cohn Fraction V (BSA), pH 7.0 were from SERVA (Heidelberg, Germany). The WesternBright™ Quantum detection kit for the chemiluminescent detection and the Western Blot Strip-it Buffer were from Advansta (Menlo Park, CA, USA).

Bilastine was obtained by dissolution of the commercial drug Robilas^®^ (A. Menarini, Industries Farmaceutiche Riunite s.r.l., Florence, Italy) with *N*-methyl-pyrrolidone (final concentration 0.1%), a solubilizer with low toxicity both orally and parenterally (Solubility Improvement of Drugs using *N*-Methyl Pyrrolidone).

### 4.2. Animal Care and Ethics Statement

Five six-week-old male DBA2/J mice (Charles River Laboratories, Calco, Italy) were maintained in compliance with the European Council directives (No. 2010/63/EU) and with the Principles of Laboratory Animal Care (NIH No. 85-23, revised 2011). The animals were kept at constant environmental and nutritional conditions at 25 ± 2 °C with alternating 12 h light and dark cycles and fed with a standard diet during a 1-week adaptation period. They were fed with a standard pellet diet (Piccioni, Settimo Milanese, Milan, Italy) and watered ad libitum. The scientific project was approved by the Ethical Committee of Turin University and by the Italian Ministry of Health (Authorization No. 279/2016 PR, approval date: 17/03/2016). The minimum sample size of 10 animals/group was determined by applying the Fleiss test for an unmatched case-control study as power analysis. The confidence interval was 90%, the power was at 85%, and the alpha level was set at 0.05. This design provides the power to investigate the differences in renal function between the different groups.

### 4.3. Experiment Protocol

Diabetes was induced in DBA2/J 18.8–20.7 g mice by a multiple low-dose STZ-intraperitoneal injection (50 mg/kg per day STZ freshly made in 0.1 mol/L citrate buffer, pH 4.5) for 5 consecutive days. Control animals were treated with vehicle alone (Figure 1). Diabetes was defined as fasting blood glucose level ≥200 mg/dL, and the onset of diabetes was evaluated by measuring 6 h fasting blood glucose using a Glucocard MX Blood Glucose Meter. After onset of diabetes, the selective H_1_ receptor antagonist bilastine was administered daily for 15 weeks as a water solution by oral gavage at 1, 3, 10, 30 mg/kg (Figure 1). Weight, food, and water intake were reordered on a weekly basis. At the end of the experimental period, mice were anaesthetized with isoflurane and killed by cardiac exsanguination. Blood and kidneys were collected for biochemical and morphological analyses on renal function. Data recording and data analysis were blinded to both the operators and the analysts, with only the individual administered the drug aware of the drug treatments given. Animal specimens were randomly labelled by a unique numeric code by A.C.R., to guarantee blind tissue sample processes.

### 4.4. Renal Function Evaluations

Twenty-four hour urine collection was performed using metabolic cages. Urine volume and pH were determined. UPE was measured by Bradford method using Bovine Serum Albumin as the standard. Albuminuria was determined by ELISA. Creatinine was measured on both plasma and urine samples by a High-Performance Liquid Chromatography (HPLC) reverse-phase method as previously described [15].

### 4.5. Morphological Analysis

Kidneys specimens were fixed by immersion in 4% paraformaldehyde, 0.1 M phosphate buffered saline (PBS) pH 7.4 overnight, embedded in paraffin. Therefore, the specimens were cut in 5 µm thick sections. Hematoxylin and eosin staining were carried out in order to analyze the gross tissue organization, May–Grunwald–Giemsa staining to evaluate leukocyte infiltration, while PAS reaction (0.5% Periodic Acid Solution) was performed to quantify mesangial matrix expansion. Moreover, renal fibrosis was assessed by picrosirius red staining, a reliable and sensitive method for the quantitative evaluation of collagen fibers [38], carried out using 0.1% picrosirius red. The morphometrical measurements of PAS and picrosirius red stained sections were examined and pictures were acquired with Zeiss Axioskop microscope (Zeiss, Mannheim, Germany). In particular, 20 microscopical fields/specimen were randomly selected and were digitalized at 100× and 20× magnifications for PAS and picrosirius red, respectively. Data analysis and measurements were performed with ImageJ software (version 1.48v; National Institutes of Health, Bethesda, MD, USA).

### 4.6. Transmission Electron Microscopy

Renal biopsies were cut in 1 mm^3^, fixed at 4 °C in 4% glutaraldehyde (phosphate buffered, pH 7.2), post-fixed in 1% osmium tetroxide and embedded in Epon 812 using gelatin capsules. Semi-thin sections were obtained with an LKB NOVA ultra-microtome (Stockholm, Sweden), stained with a solution of toluidine blue in 0.1 mol/L borate buffer, and observed under a light microscope to check the area of interest selecting at least three renal glomeruli per biopsy. Ultrathin sections were stained with Uranyless (Electron microscopy sciences, Hatfield, PA, USA) and alkaline bismuth sub-nitrate and then examined under a JEM 1010 electron microscope (Jeol, Tokyo, Japan) at 80 kV.

### 4.7. Immunofluorescence Analysis

NHE3 immunoreactivity was determined on 5 µm thick tissue sections. The sections were deparaffinized and re-hydrated, followed by microwave antigen retrieval in 10 mM sodium citrate, pH 6.0. In order to quench the autofluorescence and to minimize the non-specific binding, sections were incubated in 2 mg/mL glycine for 10 min and then for 20 min at room temperature with 1.5% bovine serum albumin in PBS pH 7.4. Sections were subsequently incubated overnight with rabbit polyclonal anti-NHE3. The immunoreactions were revealed by incubation with donkey anti-rabbit Fluor 594-coniugated IgG for 2 h at room temperature. Negative controls were carried out by omitting the primary antiserum. The immunoreaction products were observed, and pictures were acquired with Apotome systems (Zeiss) using 63× magnification.

### 4.8. Immunoblotting

Kidney randomly selected from 5 animals/group were lysed in cold buffer (10 mM Tris/HCl pH 7.4, 10 mM NaCl, 1.5 mM MgCl_2_, 2 mM Na_2_ EDTA, 1% Triton X-100), supplemented with 10× Sigmafast Protease Inhibitor cocktail tablets. Total protein content was measured spectrophotometrically using a micro-BCA™ Protein Assay Kit. Forty micrograms of total proteins were randomly electrophoresed by SDS-PAGE and blotted onto PVDF membranes. The membranes were incubated overnight at 4 °C with rabbit polyclonal anti-podocin, P-cadherin, and NHE3 or with goat polyclonal anti-nephrin and anti-synaptopodin. The rabbit polyclonal anti β-actin antibodies were used as a control. The bands were detected using rabbit or goat peroxidase-labelled secondary antibody and enhanced by WesternBright™ Quantum detection kit. Chemioluminescence signal was captured by the CDD camera ChemiDoc^TM^ (Bio-Rad, Segrate, Italy) or, alternatively, by X-ray film exposure. The densiometric analysis was performed by ImageJ software.

### 4.9. Statistical Analysis

Data were reported as mean values (± standard error of the means, S.E.M.). Statistical analysis was performed using one-way analysis of variance (ANOVA). PostHoc Calculations applying the Tukey’s multiple comparisons test were made with Prism 6 statistical software (GraphPad Software, Inc., San Diego, CA, USA). Significance was set at probability value (*p*) of < 0.05.

## 5. Conclusions

In conclusion, our data strongly support the hypothesis that H_1_ receptor could contribute to the glomerular damage occurring in DN. Bilastine was able to preserve, at least partially, the junctional integrity of the SD, through both direct and indirect effects on podocyte cytoarchitecture. Therefore, there is some scope to hypothesize the use of anti-H_1_ receptor antagonists as add-on therapy for DN, however a comparative analysis of different anti-H_1_ antihistamines in DN is still needed.

## Figures and Tables

**Table 1:** Renal function parameters at week 14 after diabetes onset.

	CTRL	CTRL + Bilastine 30 mg/kg	STZ	STZ + Bilastine (mg/kg)			
				1	3	10	30
Glucosuria ° (mg/dL)	n.d.	n.d.	1500 ± 224	1000 ± 0	1188 ± 188	1143 ± 143	1500 ± 289
Urine volume (mL)	6.8 ± 0.7	6.4 ± 0.7	19.8 ± 1.1 *	22.5 ± 2.9 *	17.5 ± 3.6 *	21.6 ± 2.3 *	32.5 ± 2.5 *
Leukocyte	-	-	-	-	-	-	-
Urine pH °	6.5 ± 0.0	6.4 ± 0.1	6.0 ± 0.2	6.0 ± 0.2	6.2 ± 0.1	6.0 ± 0.1	5.9 ± 0.1
UPE (mg/mL)	0.8 ± 0.1	0.8 ± 0.1	3.2 ± 0.2 *	3.7 ± 0.5 *	2.0 ± 0.4	2.8 ± 0.4 *	3.6 ± 1.1 *
ACR (µg/mg)	66.9 ± 6.0	60.4 ± 9.7	257.4 ± 27.2 *	150.6 ± 33.8	101.4 ± 23.1	132.2 ± 9.8	90.3 ± 40.9 ^#^
CrCl (mL/min)	0.13 ± 0.01	0.14 ± 0.01	0.05 ± 0.01 *	0.05 ± 0.01 ^§^*	0.09 ± 0.03	0.14 ± 0.01 ^§#^	0.15 ± 0.02 ^#^

° = semi-quantitative analysis by dip-stick; UPE = Urinary Protein Excretion; ACR = Albumin-to-Creatinine Ratio; CrCl = Creatinine Clearance; * vs. CTRL *p* < 0.05; # vs. STZ; *p* < 0.05; ^§^ vs. STZ + 30 mg/kg; *p* < 0.05; n.d. = under detection limit; - = negative.

## Abbreviations

ESRD	End-Stage Renal Disease
ACR	Albumin-to-Creatinine Ratio
GBM	Glomerular Basement Membrane
IP3	inositol 1,4,5-trisphosphate
NHE	Sodium-Hydrogen Exchanger
PAS	Periodic Acid-Schiff
PKC	Protein Kinases C
STZ	Streptozotocin
UPE	Urinary Protein Excretion
CrCl	Creatinine Clearance
DN	Diabetic Nephropathy
FP	Foot Processes
SD	Slit Diaphragm
ZO	Zonula Occludens
